# Supplementary material for: “Until I Know It’s Safe for Me”: The Role of Timing in COVID-19 Vaccine Decision-Making and Vaccine Hesitancy
Source: Vaccines (Basel). 2021 Nov 30;9(12):1417. doi: 10.3390/vaccines9121417 (PMC8705559; doi:10.3390/vaccines9121417)
Supplement: Supplementary file 1 [file vaccines-09-01417-s001.zip › vaccines-1409331-supplementary.pdf]

*Supplementary Material*

*"Until I Know It's Safe for Me": The Role of Timing in COVID-19 Vaccine Decision-Making and Vaccine Hesitancy*

**Eric B. Kennedy[1,*], Jean-François Daoust[2], Vivian Nelson[3], and Jenna Vikse[3]**

1. Disaster and Emergency Management, York University, Canada
2. Politics & International Relations, University of Edinburgh, United Kingdom
3. Discourse, Science, Publics Lab, Department of Psychology, University of Guelph, Guelph

* Correspondence: ebk@yorku.ca

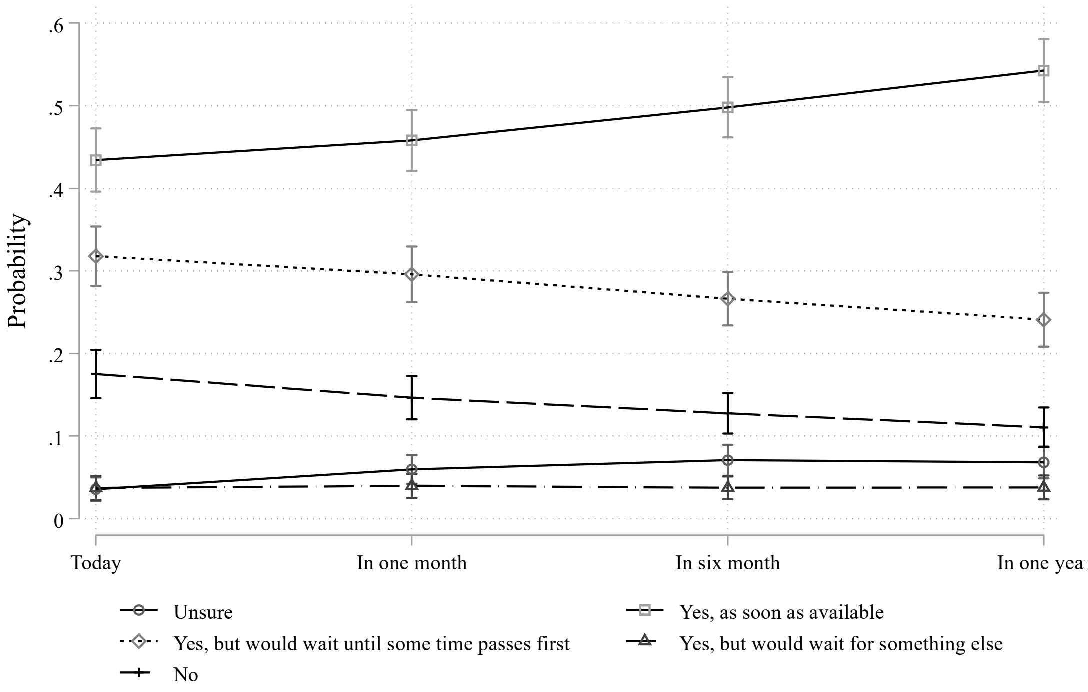


**Figure S1.** Including respondents who answered 'Unsure'.
Note: Estimations obtained from Table S1. 95% confidence intervals included.

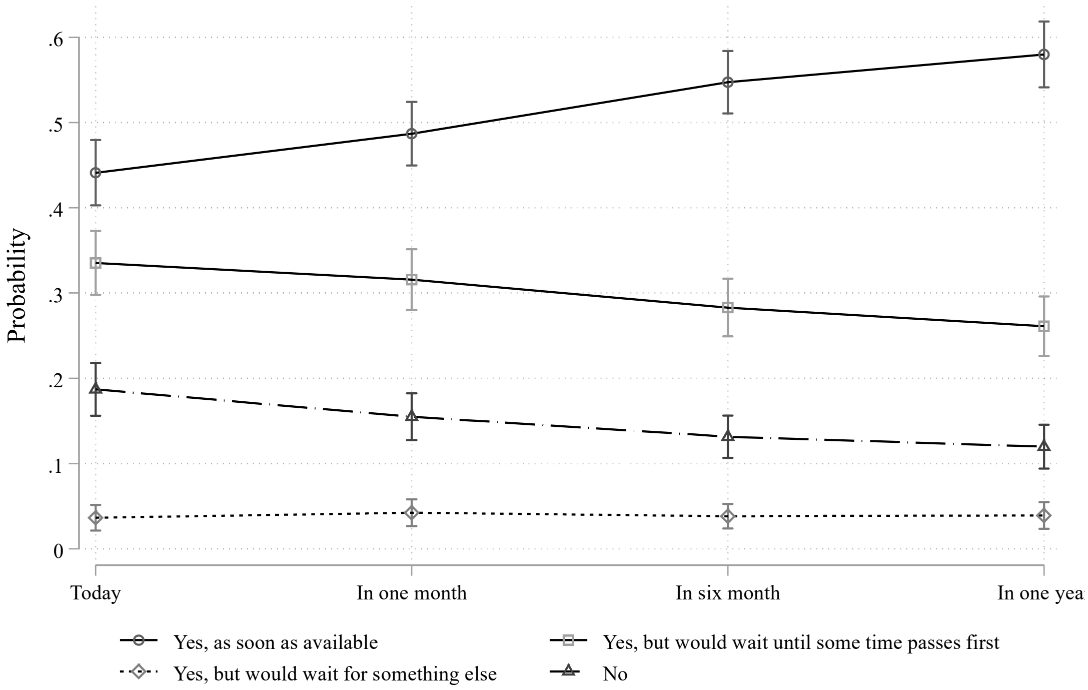


**Figure S2.** Controlling for covariates (age, gender, education, province, francophone)

**Table S1.** Multinomial logistic regression with controls

| | Yes… Until some time | Yes… Something… | No |
|---|---|---|---|
| In one month | -0.17 | 0.04 | -0.31 |
| | (0.13) | (0.30) | (0.17) |
| In six month | $-0.41^{**}$ | -0.20 | $-0.61^{***}$ |
| | (0.13) | (0.30) | (0.17) |
| In one year | $-0.56^{***}$ | -0.24 | $-0.78^{***}$ |
| | (0.14) | (0.31) | (0.18) |
| Age: 25-34 | -0.02 | 0.73 | $0.55^{*}$ |
| | (0.19) | (0.44) | (0.24) |
| Age: 35-44 | -0.02 | 0.75 | $0.51^{*}$ |
| | (0.19) | (0.43) | (0.24) |
| Age: 45-54 | -0.23 | -0.08 | 0.12 |
| | (0.18) | (0.45) | (0.23) |
| Age: 55-64 | $-0.43^{*}$ | -0.70 | -0.38 |
| | (0.18) | (0.50) | (0.24) |
| Age: 65+ | $-1.05^{***}$ | -0.45 | $-1.24^{***}$ |
| | (0.18) | (0.45) | (0.26) |
| Gender (female=1) | $0.19^{*}$ | 0.02 | $0.50^{***}$ |
| | (0.09) | (0.21) | (0.12) |
| Post-secondary | 0.07 | 0.07 | $-0.40^{*}$ |
| | (0.13) | (0.30) | (0.16) |
| University degree | -0.19 | -0.12 | $-0.92^{***}$ |
| | (0.12) | (0.28) | (0.16) |
| Quebec | 0.08 | -0.45 | 0.10 |
| | (0.24) | (0.63) | (0.31) |
| Eastern provinces | $-0.53^{***}$ | -0.51 | $-0.62^{**}$ |
| | (0.16) | (0.37) | (0.22) |
| Manitoba | -0.44 | -0.09 | -0.04 |
| | (0.23) | (0.46) | (0.27) |
| British Columbia | $-0.43^{**}$ | -0.08 | -0.05 |
| | (0.16) | (0.33) | (0.20) |
| Saskatchewan | $-0.73^{**}$ | -0.35 | -0.48 |
| | (0.24) | (0.50) | (0.30) |
| Alberta | $-0.52^{**}$ | $-0.90^{*}$ | -0.16 |
| | (0.17) | (0.45) | (0.21) |
| Francophone | $-0.71^{**}$ | 0.03 | -0.61 |
| | (0.25) | (0.65) | (0.33) |
| Constant | $0.40^{*}$ | $-2.24^{***}$ | -0.31 |
| | (0.20) | (0.48) | (0.25) |
| Observations | 2503 | | |
| Pseudo $R^2$ | 0.048 | | |

Note: Multinominal logistic regression coefficients with standard errors in parentheses. The base outcome is “Yes, as soon as possible.” The reference category for the treatments is “Today.” $^{*}p < 0.05$, $^{**}p < 0.01$, $^{***}p < 0.001$

**Table S2.** Comparison between survey sample and Canadian census

| | 2016 Canadian census | Survey sample |
|---|---|---|
| **Gender** | | |
| Women | 50.9% | 52% |
| Men | 49.1% | 48% |
| **Age** | | |
| 18-24 | 10.9% | 10.2% |
| 25-34 | 16.4% | 15% |
| 35-44 | 16.2% | 16.7% |
| 45-54 | 17.9% | 20.2% |
| 55-64 | 17.5% | 17.5% |
| 65+ | 21.1% | 20.5% |
| **Education** | | |
| Postsecondary certificate, diploma or degree | 55.3% | 48.6% |
| **Province** | | |
| Ontario | 38.3% | 34.4% |
| Quebec | 23.2% | 20.9% |
| British Columbia | 13.2% | 12% |
| Alberta | 11.6% | 10.7% |
| Manitoba | 3.6% | 5.2% |
| Saskatchewan | 3.1% | 4.7% |
| Eastern provinces | 6.6% | 12.2% |

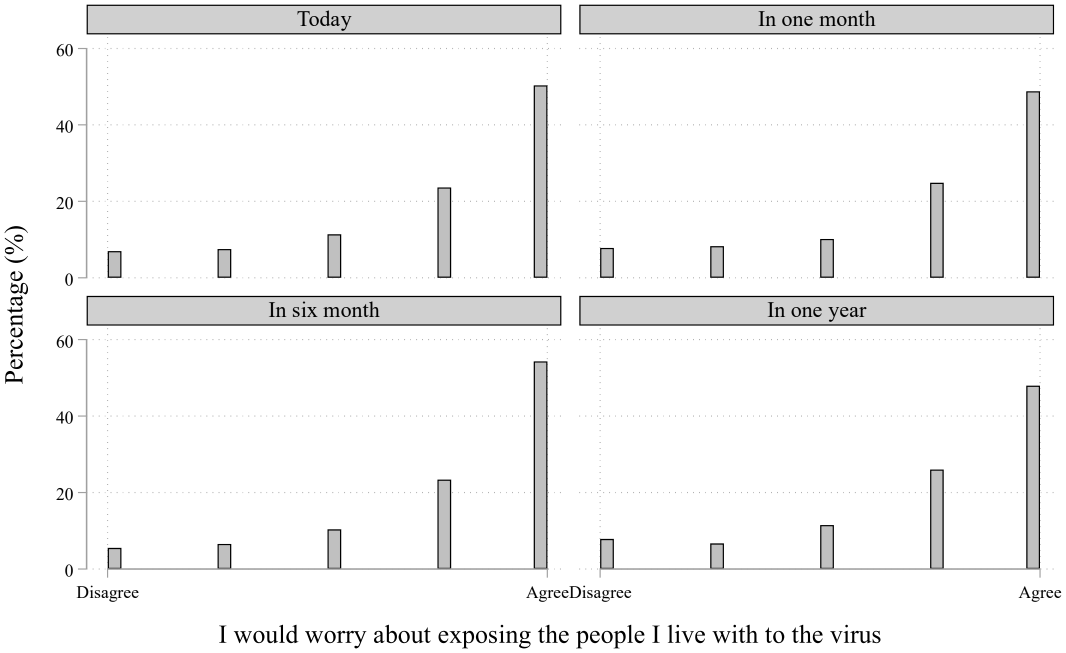


**Figure S3.** Distribution of 'worry about risks for family' across treatments

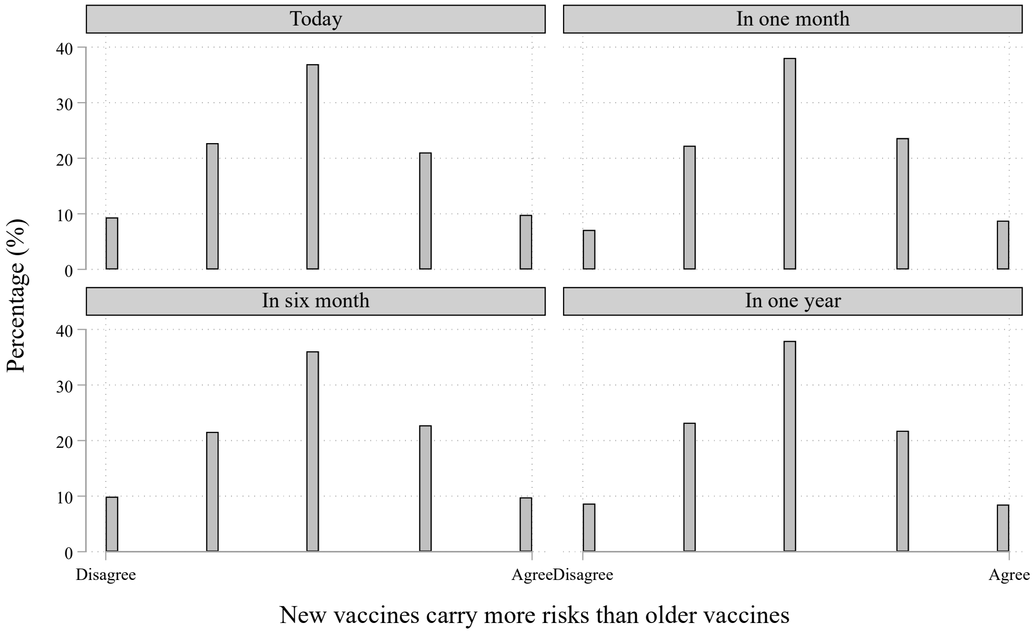


**Figure S4.** Distribution of 'risk perceptions of vaccines' across treatments.
*Note: N=2,558*

There were four treatments, each of them having a 0.25 probability to be assigned to a respondent. Treatments one and four are shown as examples of the way the question changed (substituting “today,” “in one month,” “in six months,” and “in one year”). Then, treatment two (“in one month”) is shown to demonstrate how the open-ended text boxes would appear if participants selected one of the two “would wait” options.

(a) Treatment 1 – “today”

If a coronavirus vaccine was available to you today, would you get vaccinated, or not?

- ○ Yes, I would get a vaccination as soon as one became available to me
- ○ Yes, I would get a vaccination, but would wait until some time passes first
- ○ Yes, I would get a vaccination, but would wait for something else
- ○ No, I would not get a coronavirus vaccination
- ○ Prefer not to say
- ○ Unsure

(b) Treatment 4 – “one year”

If a coronavirus vaccine was available to you in one year, would you get vaccinated, or not?

- ○ Yes, I would get a vaccination as soon as one became available to me
- ○ Yes, I would get a vaccination, but would wait until some time passes first
- ○ Yes, I would get a vaccination, but would wait for something else
- ○ No, I would not get a coronavirus vaccination
- ○ Prefer not to say
- ○ Unsure

(c) Treatment 2, illustrating the pop-up text field that would appear for any participant in any treatment who selected the option "Yes, I would get a vaccination but would wait until some time passes first".

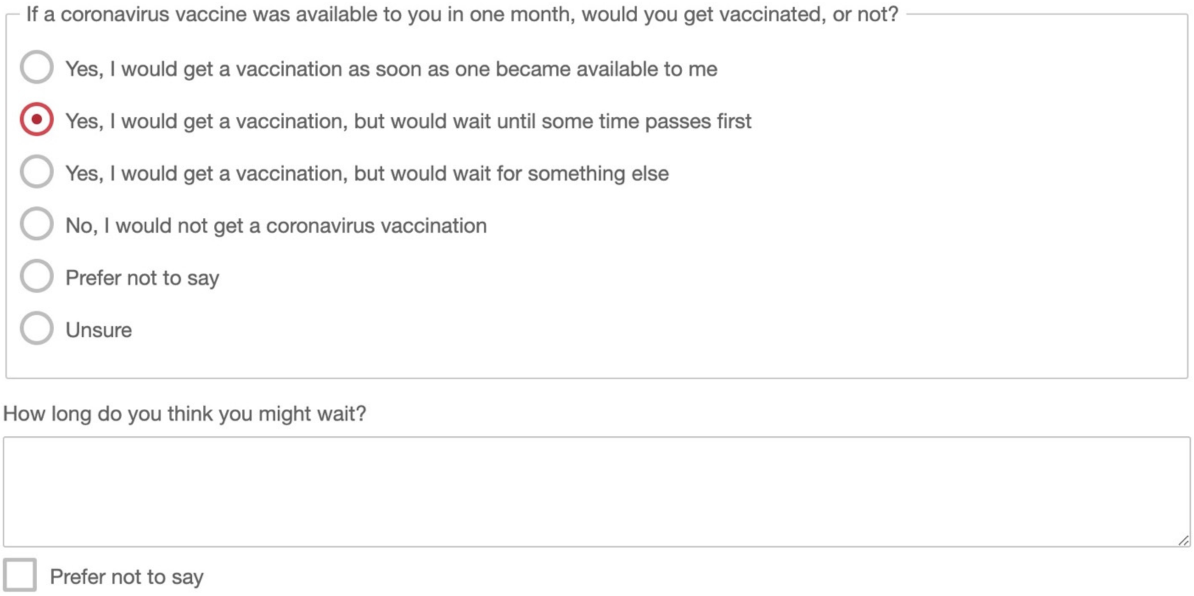
If a coronavirus vaccine was available to you in one month, would you get vaccinated, or not?

- Yes, I would get a vaccination as soon as one became available to me
- Yes, I would get a vaccination, but would wait until some time passes first
- Yes, I would get a vaccination, but would wait for something else
- No, I would not get a coronavirus vaccination
- Prefer not to say
- Unsure

How long do you think you might wait?

Prefer not to say

(d) Treatment 2, illustrating the pop-up text field that would appear for any participant in any treatment who selected the option "Yes, I would get a vaccination but would wait for something else".

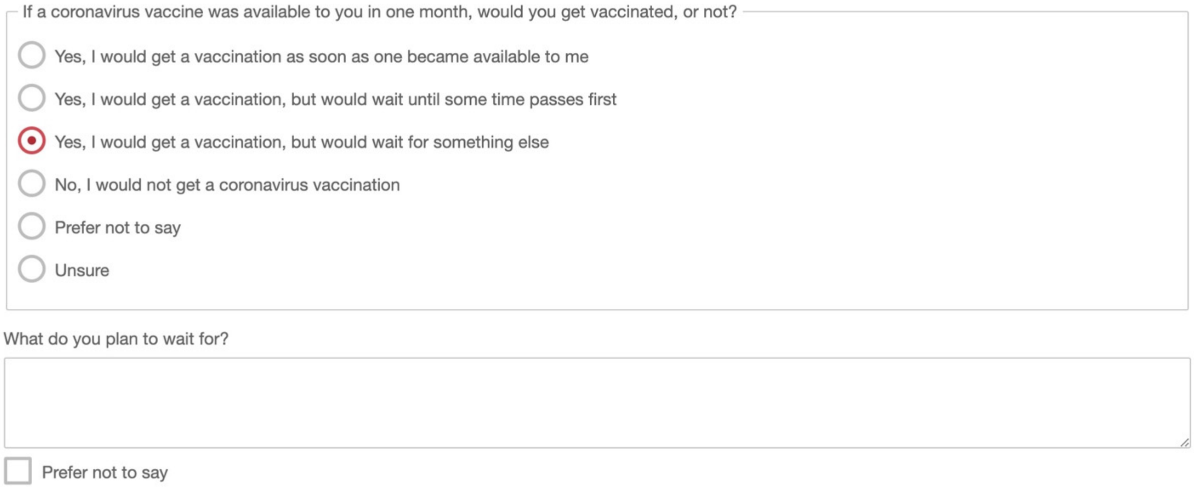
If a coronavirus vaccine was available to you in one month, would you get vaccinated, or not?

- Yes, I would get a vaccination as soon as one became available to me
- Yes, I would get a vaccination, but would wait until some time passes first
- Yes, I would get a vaccination, but would wait for something else
- No, I would not get a coronavirus vaccination
- Prefer not to say
- Unsure

What do you plan to wait for?

Prefer not to say

**Figure S5a, S5b, S5c, S5d.** A visual overview of the survey experiment
